# Clinical and genetic analysis in a patient with primary renal glucosuria: Identification of a novel mutation in the *SLC5A2* gene

**DOI:** 10.3892/etm.2013.1326

**Published:** 2013-10-04

**Authors:** YONG-WHA LEE

**Affiliations:** Department of Laboratory Medicine and Genetics, Soonchunhyang University Bucheon Hospital and Soonchunhyang University College of Medicine, Bucheon, Gyeonggi 420-767, Republic of Korea

**Keywords:** renal glucosuria, *SLC5A2*, SGLT2, mutation, Korea

## Abstract

Primary renal glucosuria (PRG; OMIM #233100) is characterized by persistent glucosuria due to a reduction in the renal tubular reabsorption of glucose in the presence of a normal concentration of serum glucose and the absence of any other impairment of tubular function. The *SLC5A2* gene is the causative gene, which codes for the low-affinity sodium/glucose co-transporter SGLT2. In the present study, the case of a patient with PRG associated with a novel mutation of the *SLC5A2* gene is reported. The patient visited hospital for the evaluation of glucosuria in the absence of hyperglycemia, a condition that had been present for >20 years. The patient showed a fasting blood sugar level of 104 mg/dl, a 2-h postprandial sugar level of 101 mg/dl, a sodium level of 144 mmol/l, a potassium level of 3.7 mmol/l and a chloride level of 106 mmol/l in serum. Urine chemistry revealed that the amount of glucose excreted was 10.8 g/1.73 m^2^/24 h; however, the levels of the other parameters were unremarkable. Polymerase chain reaction (PCR) sequencing analysis of the *SLC5A2* gene from the patient revealed a novel 1 bp deletion mutation, which altered the coding sequence of exon 10 in the transmembrane domain (c.1162delG; Ala388ProfsX48), suggesting an autosomal dominant inheritance pattern. This study identified a novel mutation in the *SLC5A2* gene related to a benign clinical characteristic and suggests that the molecular diagnosis of the *SLC5A2* gene may be useful for diagnosing renal glucosuria in patients and for deciding intervention measures for their family members.

## Introduction

Primary renal glucosuria (PRG; OMIM #233100; http://omim.org/entry/233100. Accessed August 5, 2012) is characterized by persistent glucosuria due to a reduction in the renal tubular reabsorption of glucose in the presence of a normal concentration of serum glucose and the absence of any other impairment of tubular function. It is caused by mutations in the *SLC5A2* gene on chromosome 16p11.2, which codes for the low-affinity high-capacity sodium/glucose co-transporter SGLT2 (1). This transporter is responsible for the tubular reabsorption of filtered glucose. Numerous case reports have confirmed that *SLC5A2* mutations are responsible for the majority of cases of PRG (2–7).

To date, >50 different mutations of the *SLC5A2* gene have been reported, the majority of which are restricted to a single individual or family. Although PRG is known to be inherited as an autosomal recessive trait, certain cases of PRG have demonstrated co-dominance or a pattern of autosomal dominance. Numerous heterozygous individuals have mild glucosuria, whereas homozygous or compound heterozygous mutations normally have massive glucosuria >10 g/1.73 m^2^/24 h. Co-dominance with incomplete penetrance supports the best fit for the inheritance pattern of PRG (2).

In the current study, the clinical and genetic findings of a subject with PRG associated with a novel mutation of the *SLC5A2* gene are described. The well-being of the patient suggests that PRG may have a benign nature.

## Case report

The subject of this study is a healthy 40-year-old man, the fifth child of Korean parents. The parents of the patient have no history of glucosuria. The patient had exhibited glycosuria in the absence of hyperglycemia for >20 years and attended Soonchunhyang University Bucheon Hospital (Bucheon, Korea) for an evaluation. The patient was noted to have glucosuria at a routine urinalysis check-up. The patient had no history of diabetes or hypertension, or of cardiac, pulmonary, hepatic, renal or musculoskeletal disorders, and exhibited no evidence of hypoglycemia. Furthermore, the patient exhibited no urological manifestations related to renal glucosuria. Work-ups repeated in order to elucidate the cause of the persistent glucosuria failed. The patient reported that two older brothers had a history of glucosuria.

An extensive laboratory work-up was performed in order to investigate the cause of the patient’s renal glucosuria. Urine amino acid analysis, 24-h urine chemistry and urine osmolality were performed.

Molecular defects in the *SLC5A2* gene were investigated to confirm the diagnosis of PRG. After obtaining informed consent from the patient, blood samples were collected. Genomic DNA was isolated from peripheral blood leukocytes using a Wizard genomic DNA purification kit according to the manufacturer’s instructions (Promega, Madison, WI, USA). The *SLC5A2* gene was amplified by polymerase chain reaction (PCR) using primers designed by the authors (forward: 5′-ACAACGGTCTAAGGCGCAGTC-3′, reverse: 5′-TTAGGAGGGTGACGGAACTGG-3′) and a Thermal Cycler 9700 (Applied Biosystems, Foster City, CA, USA). Sequence analysis of all coding exons and the flanking introns of the *SLC5A2* gene were performed using the BigDye Terminator Cycle Sequencing Ready Reaction kit (Applied Biosystems) on an ABI Prism 3130 genetic analyzer (Applied Biosystems). Nucleotide numbering reflects cDNA numbering with c.1 corresponding to A of the ATG translation initiation codon in the reference sequence of SLC5A2 (NM_003041.3). Potential mutations were defined by their exclusion from the Human Gene Mutation Database (http://www.hgmd.cf.ac.uk) and previously reported mutations on PubMed (http://ncbi.nlm.nih.gov/PubMed/). All novel mutations were confirmed by sequencing 100 control chromosomes. The study was approved by the ethics committee of the Institutional Review Board and also the screening of the SLC5A2 gene in normal subjects was approved by the ethics committee of Soonchunhyang University Bucheon Hospital (Bucheon, Korea).

## Results

The patient exhibited a fasting blood sugar level of 104 mg/dl [reference range (RR), 60–108 mg/dl], a 2-h postprandial sugar level of 101 mg/dl, a sodium level of 144 mmol/l (RR, 135–145 mmol/l), a potassium level of 3.7 mmol/l (RR, 3.5–5.5 mmol/l), and a chloride level of 106 mmol/l (RR, 98–110 mmol/l) in serum. Twenty-four-hour urine chemistry revealed that the quantity of glucose excreted was 10.8 g/1.73 m^2^/24 h (RR, <0.5 g/1.73 m^2^/24 h). However, protein, uric acid, urea nitrogen, creatinine, sodium, potassium, chloride, calcium and phosphorus levels were within the RR. The osmolality of urine was 763 mOsm/kg (RR, 300–900 mOsm/kg), and amino acid analysis in urine revealed no amino aciduria. There was no evidence of proteinuria, acidosis, β-2 microglobulinuria, acidosis or phosphaturia.

**Figure 1:**
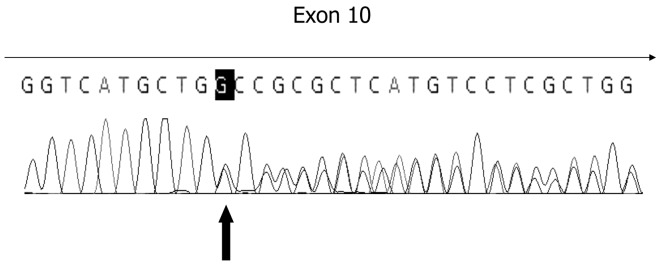
Mutation analysis of the *SLC5A2* gene from a patient with renal glucosuria restricted to a single individual or family. Direct sequencing of exon 10 reveals overlapped peaks (arrow) from nucleotide position 1162 due to a 1 bp deletion (c.1162delG; Ala388ProfsX48).

Direct sequencing of the *SLC5A2* gene from the patient with PRG revealed a novel 1 bp deletion mutation, altering the coding sequence of exon 10 in the transmembrane domain (c.1162delG; Ala388ProfsX48; Fig. 1). The 1 bp deletion results in the frameshift mutation starting from 388th codon, which is truncated at the 48th codon from Ala388. Screening of the *SLC5A2* gene in 50 normal control subjects revealed no mutant alleles in 100 screened chromosomes. *SLC5A2* gene analysis suggested an autosomal dominant inheritance pattern.

## Discussion

In this study, a novel deletion mutation in the *SLC5A2* gene in a patient with PRG, associated with benign clinical characteristics, is reported. The patient appeared healthy despite having had glucosuria since childhood and exhibited no ill effects of the glucosuria.

We speculate that glucosuria caused by carrying the *SLC5A2* gene mutation may have a potentially beneficial effect on the healthy state. The persistent loss of calories and sodium may have a beneficial effect on lowering blood pressure and preventing obesity, although the majority of the sodium is reabsorbed by distal tubules (8). A pharmacological inhibitor of SGLT2 (sodium-glucose co-transporters inhibitor) has been evaluated as a useful drug for the treatment of obesity, diabetes, and hypertension with no significant toxic effects (9).

Among sodium/glucose transporters, SGLT2 is expressed almost exclusively in the kidney (10) and is encoded by the *SLC5A2* gene, which is responsible for PRG. A large number of heterogeneous mutations have been reported in the *SLC5A2* gene. However, previous reports have not observed an identifiable mutation in the *SLC5A2* gene (2,3). This may be due to technical limitations related to mutational analysis. According to the previous reports, no clear association has been identified between genotype and phenotype in PRG, with the exception of a tendency for increased homozygous or compound heterozygous mutation frequency in patients exhibiting severe glucosuria.

Although PRG is a rare disease, many subjects with glucosuria without hyperglycemia have been confirmed by *SLC5A2* gene analysis, as in the current case. In the present case, the c.1162delG mutation affects exon 10, including an alanine residue conserved in the entire transporter superfamily to which SGLT2 belongs, abolishing the expression of the glucose transporter. This finding is in agreement with previous studies, suggesting that *SLC5A2* mutations have marked allelic heterogeneity, and the majority of mutations in PRG are restricted to a single individual or family (11).

Unfortunately, it was not possible to perform a family study; therefore, an evaluation of the mutational pattern of the patient to determine whether the mutation was *de novo* or inherited could not be conducted. Furthermore, it was not possible to induce information regarding the genotype-phenotype correlation, due to there only being one case. Despite the large deletion involving the *SLC5A2* gene, the majority of mutations may be detected by sequencing analysis. As previously reported, compound heterozygotes that carry missense or truncated mutations consistently exhibited renal glucosuria due to SGLT2 dysfunction (2–7). Therefore, identification of the mutation that causes glucosuria may enable the establishment of a genotypic diagnosis of PRG, providing important information to families and physicians.

In conclusion, PRG should be considered when making a differential diagnosis of patients exhibiting unexplained renal glucosuria without hyperglycemia or hypoglycemia, and *SLC5A2* gene analysis should be performed. Molecular diagnosis may be beneficial for the confirmation of renal glucosuria and in deciding interventional measures for the patient’s family members.
